# Supplementary material for: Survival analysis of parking duration in urban commercial areas: A case study in Zhengzhou, China
Source: PLoS One. 2025 Jun 6;20(6):e0323207. doi: 10.1371/journal.pone.0323207 (PMC12143495; doi:10.1371/journal.pone.0323207)
Supplement: Attachment — Submitted filename: Response to reviewers2.docx [file pone.0323207.s004.docx]

**Responses (R) to Reviewers’ Comments (C)**

First of all, thanks a lot for the reviewers’ advices. These comments are very valuable and helpful for improving our article. According to the reviewers’ comments, we have carefully modified our manuscript to meet the requirements of ***PLoS One.***

**Reviewer: 1**

**C: *The manuscript has improved a lot. It is better to add quantitavie research results in the abstract. The authors should do a thorough proofreading of the entire manuscript before publication.***

**R:**Thanks a lot for the reviewers' affirmation and suggestions on our paper.We highly agree with the reviewer's suggestion to include quantitative research results in the abstract and have accordingly supplemented the abstract with relevant quantitative research findings to enhance its informativeness and appeal. The relevant portion of the revised abstract is:”the parking duration is significantly influenced by weekdays (regression coefficient = 0.068, hazard ratio=1.071, P < 0.001), weekends (regression coefficient = 0.042, hazard ratio=1.043,P < 0.001), moderate rain (regression coefficient = -0.089, hazard ratio=0.914, P < 0.001), and heavy rain (regression coefficient = 0.030, hazard ratio=1.030,P = 0.034 < 0.05). The results have indicated that within the study area, compared to short holidays, the parking duration on weekdays and weekends is shorter, with the probability of vehicles ending their parking increased by 7.1% and 4.3%, respectively. Under different weather conditions, compared to sunny days, parking duration is longer during moderate rain, with the probability of vehicles departing decreased by 8.6%, whereas during heavy rain, parking duration is shorter, with the probability of vehicles departing increased by 3%.”

Furthermore, we have thoroughly proofread the entire manuscript based on the suggestions, ensuring that all potential errors and inconsistencies are eliminated prior to publication. These revisions are highlighted in red font within the manuscript.

**Reviewer: 2**

**C:*The authors have fully addressed all my comments and questions. They have revised and improved the previously submitted manuscript.***

**R:**The reviewer has indicated that we have fully addressed all their comments and questions, and have revised and improved the previously submitted manuscript. We greatly appreciate the reviewer's affirmation and encouragement, which has provided us with great motivation to continue refining the paper. We will continue to polish and optimize the paper based on the reviewer's suggestions.

**Reviewer: 3**

**C:*The authors have addressed the comments of the reviewer. The reviewer recommeds the paper for publication.***

**R:**We feel deeply honored to have received the recognition and support from the reviewer. We will continue to strive to enhance the quality of our paper and look forward to successfully publishing our research findings in this esteemed journal.
